# Interprofessional and Interdisciplinary Approach from Undergraduate Health and Pre-Medical Students in Children’s Health Educational Initiative

**DOI:** 10.4172/2161-0711.1000266

**Published:** 2013-12-26

**Authors:** Sophie R Zhao, Siyuan Cao, Patrice S Lin, Jeffrey Yenor, Regina Lam, Ellen Chang, Richard Liu, Jianghong Liu

**Affiliations:** University of Pennsylvania School of Nursing, Philadelphia, PA, USA

**Keywords:** Interprofessional, Interdisciplinary, Health Education, Community Intervention

## Abstract

The importance of interprofessional training in healthcare to improve quality of care and health outcomes has been increasingly recognized. This pilot study used an interprofessional and interdisciplinary team of undergraduate health and pre-health students to establish a unique community partnership with a local elementary school in developing and implementing a nutrition/exercise educational intervention. Our results suggest that children as young as 8 years old are capable of learning new information related to the benefits of particular food groups, are capable of retaining this knowledge for 6 months, and that an intervention program as short as one hour every few months stand to make significant impact on children’s knowledge about proper nutrition and healthy lifestyles. Our results suggest the potential benefits of further expanding the short-term intervention into a longer-term community-based curriculum targeting a younger age group previously or currently practiced.. Furthermore, this pilot study suggests that undergraduate health and pre-health students can form an interprofessional and collaborative team to take an active role in the dissemination of nutrition knowledge in the community.

## Background

In recent years, interprofessional education and training have been increasingly emphasized, particularly in the healthcare field including nursing and medicine, because healthcare providers often work in collaborative teams of physicians, nurses, social workers, administrative personnel, and others [1]. Interprofessional education and training are designed to set up a continuum of learning for healthcare professionals in order to encourage collaboration, integrate knowledge, and ultimately improve patient care [1–2]. The ultimate goal is therefore to improve flexibility and maximize resources within the healthcare provider team. There is some literature, mainly in Europe, regarding interprofessional training during higher education and its effectiveness in healthcare [1,3–5]. However, there are limited studies and implementations of interprofessional healthcare education and training at the undergraduate stage of education in the United States, where undergraduate education is separate from professional healthcare education. This pilot study takes an interprofessional and interdisciplinary approach and utilizes a community partnership to address a rising health concern in children: obesity.

The prevalence of obesity has risen dramatically in developed nations over the last 20 years. This is most clearly evident in the United States, where 35.9% of adults are now considered clinically obese and 69.2% overweight [6]. Perhaps the most serious aspect of this epidemic is its impact on children [7–8]. More than 60% of children 10 years and older are or will become obese, and many obesity-related diseases previously seen only in adults - e.g. coronary artery disease, diabetes, and hypertension - are now increasingly afflicting children of much younger age [9–12]. There has also been evidence that being overweight and obese can impair healthy physicial, psychosocial, and behavioral development in children [13–16] and recent research has turned to understanding risk factors, including sleep behaviour [17] and parental factors [18], associated with childhood obesity.

An effective site for obesity prevention and community intervention can be in schools, where nearly all children are accessible to interveners who can help establish and maintain healthy eating and physical activity habits [10,19–22]. School nurses can be an effective mode of program delivery because of their unique position of being the most accessible in creating a healthy environment for children [20]. Unfortunately, constraints in scheduling and financial availability, among other challenges, have prevented such interventions from being more widely implemented [19, 23–24]. Additionally, there is a paucity of research addressing the actual effectiveness of health and pre-health students in health promotion [23,25]. Therefore, forging community partnerships appears to be a potential solution to these problems [21,26–27], as health and pre-health students, faculties, and administrators from academic institutions can step in to provide the time and resources which school nurses alone may lack. Individual studies of school-based interventions provide promising frameworks for comprehensive and appealing health education for elementary school students [21, 26].

The complex etiology of obesity suggests that the best approach for intervention might be through an interprofessional effort, which only a few previous studies have utilized. However, the time and resources likely required to cohesively integrate multiple healthcare professionals from several different specialties suggests nursing which takes perhaps the most comprehensive approach to patient education may be best-suited to delivering the message. In one recent study, teams of undergraduate nursing students and faculty members delivered the Let’s Go 5-2-1-0 Program at two different elementary schools [27]. Using this school/nursing students model, a successful community partnership was created that effectively addressed key themes, provided conceptual reinforcement for the children, empowered everyone involved, as well as overcame time constraints which often afflict school [nurses 27]. Thus, it appears health and pre-health students supervised by their faculty can provide efficient and effective adjuncts to school nurses in the health education of school-age children.

However, there is still a relative lack of evidence in the literature addressing how exactly interprofessional undergraduate pre-health students can fit into community-based childhood obesity intervention programs. Furthermore, most relevant studies focus on older elementary school children and adolescents (ages 10 and above) or parents and their infants [28–29]. Only recently have researchers turned their attention to younger elementary school children’s existing knowledge of nutrition and active lifestyle and their ability to effectively learn this material in a school setting [30–31]. In addition, while there exists the CATCH Program that stems from a study involving third graders, it lacks the presence of nurses, health and pre-health students to relay the information; we are particularly interested in how receptive young elementary school children are to health information and how able they are to retain this information disseminated by health and pre-health students [32]. Our study sought to add unique elements to the school/nursing student model by recruiting a team of interdisciplinary undergraduate pre-health students and providing a pilot nutrition and lifestyle education program in a local third-grade classroom. This experience served to enrich the elementary school and undergraduate students alike. The elementary school students had the advantage of being taught by educators closer to their age–perhaps improving their ability to relate to the presented material–and the undergraduates were given the chance to enhance their exposure and involvement in health science research directly in the community and to collaborate in an interprofessional team, opportunities not traditionally available or readily accessible to undergraduate health and pre-health students. It is our hope to eventually develop an interprofessional training curriculum for greater undergraduate health and pre-health student involvement in the community in order to more positively impact the dearth of effective nutrition and health education present in today’s school systems at all age levels.

## Materials and Methods

### Formation of the interprofessional training team

The intervention and questionnaire were created and implemented by a team of interprofessional undergraduate professional students, including nursing and pre-medical students, with interdisciplinary academic backgrounds. These students worked with a faculty member of both the Department of Community & Family Health in School of Nursing and the University of Pennsylvania Master of Public Health Program, whose research specialty includes pediatric health and nutrition. More specifically, the team of undergraduates comprised of a dual-degree student studying nursing and health care management, a dual-degree student studying biological science and business, a health and societies student focusing on public health, a pre-medical student with a background in the physical sciences, and a nursing student studying pediatrics with extensive academic training in kinesiology. Each member of this interprofessional team contributed their unique background and training to the development of this pilot intervention. The questionnaire and presentation materials were reviewed by the supervising faculty member to leverage her expertise in pediatric health and to confirm the age appropriateness of the intervention.

### Setting

The health education interventions were implemented twice; the first intervention took place in the fall of 2010 and the second in the spring of 2011 at a local public elementary school located in West Philadelphia. This school was strategically chosen for its close location to the University, since one of the main goals of this pilot study is to potentially establish future community-based partnerships and follow-up studies. A post-intervention follow-up assessment was also conducted in the summer of 2011.

### Subjects

Our study targeted two classes of English-speaking third grade students. The school’s ethnic profile for the 2010–2011 school year consisted of 36.1% African American, 31.8% Caucasian, 16.3% Asian, 7.3% Latino, and 8.5% other. Additionally, 47.3% of the student body was considered economically disadvantaged. This profile is a good representation of the diverse population in West Philadelphia. A total of 51 students participated in the first round intervention in fall 2010, 47 participated in the second round intervention in spring 2011, and 34 participated in the post-intervention follow-up in the summer of 2011. The sample was not chosen randomly. No identification information (e.g. name, gender, age) was collected from the students.

### Development of intervention

As existing literature reveals that inadequate physical activity levels and unhealthy eating habits are the two most prevalent causes of childhood obesity [9,21,24–26,33–36] we divided our intervention into two parts–the first focusing on nutrition and making healthy food choices and the second focusing on exercise and making active lifestyle choices. The intervention strived to promote better nutrition knowledge and healthy eating habits, increase children’s physical activity levels, prevent obesity, and assist in maintaining an improved lifestyle.

The developed intervention consisted of PowerPoint slides containing both words and pictures and was designed with detailed objectives in mind (Table 1). As the students involved were in their middle childhood years, we hypothesized they had the ability to group items into different subsets. This hypothesis was drawn from Jean Piaget’s cognitive development theory, which suggests third graders’ ability to use seriation and to categorize items [37]. As third graders are in the concrete operational stage of this theory, our intervention constantly required them to group items into categories. As children in this age are also expected to be able to use seriation, we utilized My Pyramid as a constant visual aid to explain to the students that the different column width of each food category corresponds to healthy relative portions.

During the development of the PowerPoint presentation, we made constant efforts to include vibrant pictures of foods and to refer to a diagram of MyPyramid [38]. The continuous referral to this diagram aimed to enhance knowledge retention, as visual presentation of information has been shown to facilitate the learning and recall of information better than auditory presentations alone [39]. Thus, while we verbally elaborated on the information presented on each slide, almost all slides comprised mainly of pictures.

### Delivery of interventions

We delivered the same intervention at two points in time, once in the fall of 2010 and again in spring 2011, 6 months later. This was done to investigate the effects of repeated intervention as opposed to that of a single intervention.

The intervention and the completion of questionnaires took place for approximately one and a half hours on two separate Friday afternoons and were scheduled during times when no academic classes were planned. The delivery of intervention consisted of three parts: 1) a two-minute frame for third graders to share their favorite foods; 2) a 30-minute PowerPoint presentation focusing on the different groups of MyPyramid and the nutritional benefits of each for the human body; and 3) a 20-minute PowerPoint presentation focusing on the importance of exercising. Throughout the intervention, we asked the students to actively participate by answering questions about recently presented slides to promote dynamic learning and repeated information processing.

**Figure 1:**
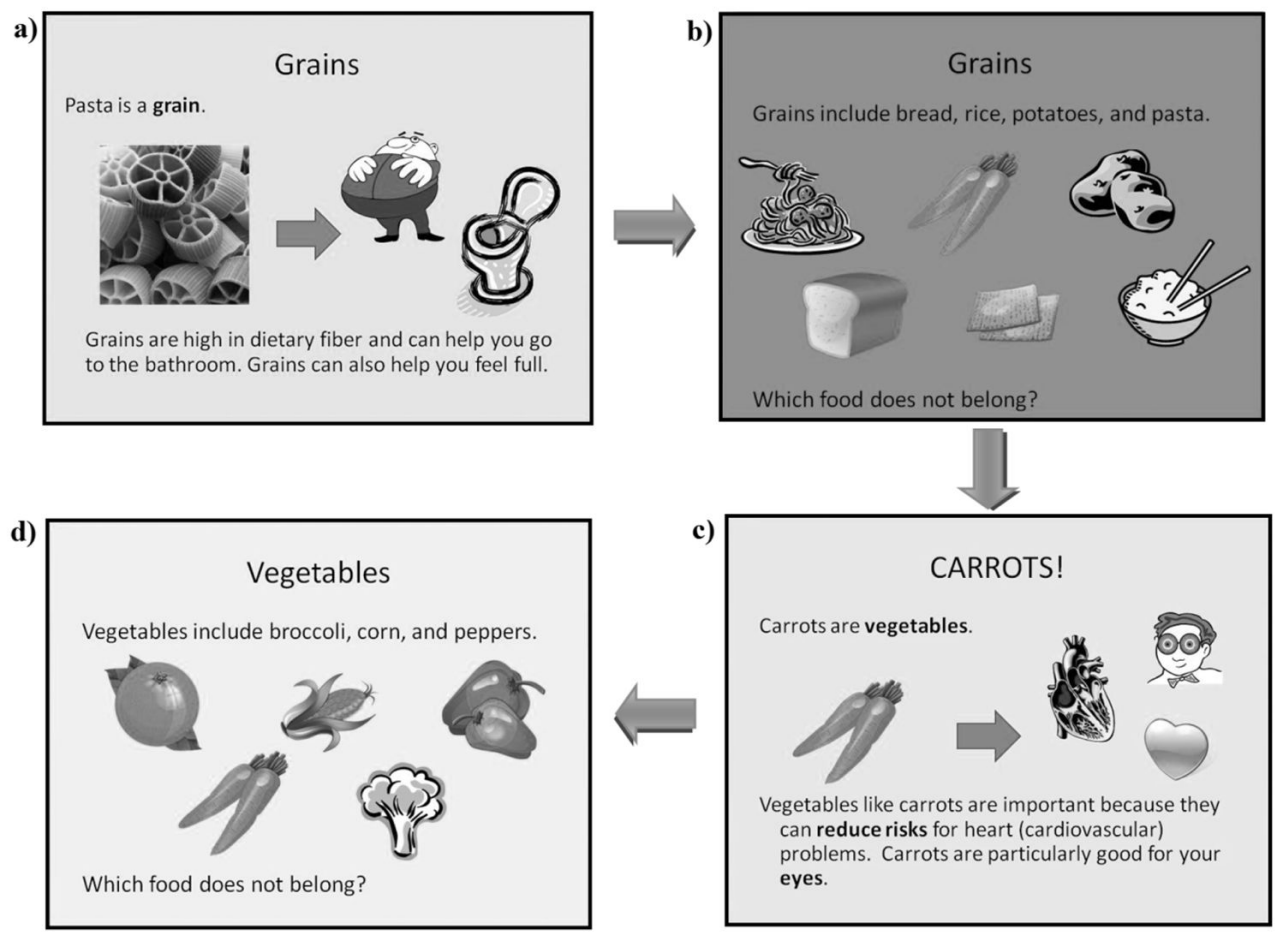
Presentation of nutrition-based slides followed a similar pattern to introduce each new food group. a) Each food group’s initial slide first introduced the food group and the nutritional benefits of foods found in the group; b) Multiple images of various foods that fit in the category were then shown. Students were asked to raise their hands to identify which one item did not belong on the slide; c) The correct food group for the misplaced item was then subsequently introduced and served as the initial slide to introduce the new food group and its nutritional benefits; d) Multiple images of various foods that fit in the new category were then shown, as in b).

The nutrition-based slides were organized by food groups and all followed a similar pattern (Figure 1). The exercise and active lifestyle portion of the intervention began with a discussion with the students about the kinds of exercise they enjoyed and why they believed exercise was beneficial for the body. After this point, the active lifestyle and exercise slides were introduced to share more explanations for the importance of exercise.

### Assessment design

We distributed paper copies of the same 15-item questionnaire at 5 points in time: before the first intervention (pre-test 1 or baseline), immediately after the first intervention (post-test 1), 6 months after the first intervention but before the second intervention (pre-test 2), immediately after the second intervention (post-test 2), and 2 months after the second intervention without any further intervention (pre-test 3). The 2 months timeframe for pre-test 3 was chosen because this was the period immediately prior to summer break for these students, after which the class would have been dispersed to prevent any future follow-ups.

The students were required to read the questions by themselves and to indicate the best answer. We announced to the students that these tests would not be graded on accuracy but simply used as an assessment of their existing knowledge and what they were able to learn from the presentation. This was done in the attempt to alleviate unnecessary pressures to score well and to minimize cheating.

Of the 15 questions, 11 focused on nutrition and 4 focused on active lifestyle and exercise. Of the 11 nutrition-focused questions, 9 were multiple-choice questions and 2 were fill-in-the-blanks. All 4 of the active lifestyle and exercise questions were multiple-choices. The only assistance students received while completing the questionnaires was from the two third-grade teachers from each respective class who clarified the meaning of “obesity.”

## Results

### Evaluation

The scores on the pre-tests and post-tests were measured as the percent of correct responses for every item on the questionnaire. The scores were broken down further for analysis based on the questions’ objective. As Table 2 shows, the questions can be categorized by two distinct objectives: nutrition and exercise/active lifestyle. We examined the responses to each question independently across all students who partook in the tests as well as composited a total score for each test and therein the average performance before and after the educational program. All statistical analyses were performed with IBM SPSS 19 [40].

### Reliability testing

Data from the group of 51 students who completed the baseline assessment was used to determine the questionnaire’s internal reliability. The reliability coefficient, as measured by Cronbach’s α, was 0.67. Since questionnaires are suggested to exhibit a reliability level of at least 0.7 [41], our questionnaire can be deemed to be acceptably reliable.

### Knowledge evaluation

To evaluate the students’ existing knowledge about nutrition and active lifestyle, the results from the baseline assessment (pre-test 1) are analyzed and key descriptive statistics are listed in Table 2. Regarding nutrition, results show that the children had the best knowledge about junk foods: 78% of the children knew that soda, fries, and chips are considered junk foods, 88% knew that French fries are unhealthy because they are high in fats and oils, and 78% knew that bacon was an unhealthy source of protein. The children’s knowledge about specific nutritional values of various food groups is more widely varied. Overall, the children showed that they had the best knowledge about the specific health benefits associated with vegetables and fruits: 57% knew that vegetables decrease heart problems, 63% knew that carrots are good for eyes, and 67% knew that oranges are high in vitamin C. Regarding knowledge about protein (i.e. macronutrients), only 20% of the children knew that meat, tofu, and beans are all good sources of proteins, but 67% knew that protein helps to build muscles. Regarding other knowledge of micronutrients, only 39% of the children recognized milk, yogurt, and cheese as all good sources of calcium and 47% knew that calcium helps build bones. However, 67% of the children did know that fish is good for the brain.

Regarding exercise and active lifestyle, the children showed generally lower levels of knowledge. While the majority knew that exercise makes the body stronger and more flexible, improves heart and lung health, and helps maintain a healthy weight, only 36% knew that exercise is good for illness prevention, better sleep, and improved school performance. Lastly, only 12% of the children were aware of the fact that obesity can be an inherited trait.

### Assessment of the results

#### First round intervention

The overall mean and standard error of the pre-test 1 and post-test 1 were, respectively, 59.07% ± 2.66% and 64.53% ± 3.61%. The mean difference was shown to be statistically different at 0.05-level (p=0.018) by a Student t-test. When the results for each objective were analyzed separately, the average performance of the portion of the test pertaining to nutrition still showed significant improvement (p=0.0004), but the portion of the test pertaining to exercise/active lifestyle no longer showed significant improvement (p=0.660). This could be due to the fact that there were so few questions on the latter objective (only 4). Missing one question would more greatly affect the average performance.

The percentages of correct responses to each of the questions during the pre- and post-test are shown in Table 3. Of the 15 questions, 7 showed significant improvement at the 0.05-level by Student t-tests; of the 8 questions that did not show significant improvement, 4 had pre-test scores of above 60%.

#### Second round intervention

The overall mean and standard error of the pre-test 1 and post-test 2 were, respectively, 59.07% ± 2.66% and 78.41% ± 2.60%. The mean difference was shown to be statistically different at 0.001-level (p<0.00001) by a Student t-test. When the results for each objective were analyzed separately, the average performance of the both portions, nutrition and exercise, showed significant improvement (p<0.00001 and p=0.00002, respectively). This indicates that the second intervention overall yielded significant improvement in all aspect of our assessment.

The percentages of correct responses to each of the questions during the pre-test 1 and post-test 2 are also shown in Table 3. At this stage, 9 out of 15 questions showed significant improvement at the 0.05-level by Student t-tests. Of the 6 questions that did not show significant improvement, 5 had pre-test scores of above 70%.

#### 6-month retention after first round of intervention

To assess the retention of knowledge after the first intervention, we analyzed the mean differences in scores between post-test 1 and pre-test 2. Pre-test 2 took place 6 months after the completion of the first round of intervention and the assessment was given without any presentation of the intervention material. The results of the comparison are shown in Table 4a.

Our analyses show that there are very good retention rates overall in the nutrition portion of the questionnaire. Although 6 out of 11 questions showed decreased odds of being answered correctly after 6 months, the decrease was found to be significant in only 1 question by Student t-tests. The exercise portion of the questionnaire actually showed significant *improvement* in performance in 3 out of 4 items by Student t-tests. In fact, all 4 items showed increased odds of being answered correctly after 6 months. This indicates that perhaps there were outside factors accounting for the children’s increased knowledge about exercise and healthy lifestyle, or perhaps it simply took time for the children to process and retain the information they were previously presented with.

#### 2-month retention after second round of intervention

To assess the retention of knowledge after the second intervention, we analyzed the mean differences in scores between post-test 2 and pre-test 3. Pre-test 3 took place 2 months after the completion of the second round of intervention and the assessment was given without any presentation of the intervention material. The results of the comparison are shown in Table 4b.

Our analyses indicate that while 13 out of 14 items showed decreased odds of being answered correctly between pre-test 3 and post-test 2, none of the decrease was found to be significant by Student t-tests.

## Discussion

This interdisciplinary and collaborative health intervention from an interprofessional team in a community setting of third graders generated several findings. We first found that the majority of third graders involved seemed to already have a general idea of which kinds of foods are considered unhealthy. In the baseline assessment, the children scored above 50% on 10 out of the 15 items on our questionnaire. The children performed particularly well in identifying foods that are unhealthy. 88% of students knew that French fries are unhealthy because of their high fats and oils composition while 78% of students could identify bacon as being the less healthy compared to green beans or eggs.

Secondly, after comparing the data from pre-test 1 to post-test 1 and to post-test 2, we found that third graders were able to understand and retain new knowledge regarding the benefits of specific foods on a short-term basis. These results suggest that third graders are cognitively capable of learning new information about the benefits of foods and food groups. Furthermore, we found that performance increased more significantly after the second-round of interventions as compared with after the first-round, pointing to the fact that compounded presentation of the same information facilitates the understanding and retention of knowledge regarding nutrition and exercise.

Lastly, our results showed that third grade children are capable of retaining the nutrition and exercise information presented in a one hour intervention on a long-term basis. Our data from 6 months post-first intervention as well as 2 months post-second intervention both indicate that the children exhibit very good retention rates in performance on our questionnaire. In fact, the performance on a number of the items on the questionnaire actually *improved* over time, without any further intervention on our parts. These findings points to the possibility that intervention programs as short as one hour every couple of months can have significant impact on children’s knowledge on nutrition, exercise, and healthy lifestyles.

Our pilot study can contribute to existing literature on childhood obesity prevention programs in several ways. First, a unique attribute of our study was the age of the sample population. While other nutrition interventions have been tested, they primarily targeted older children [15]. However, since younger children can adopt new habits more easily, it is therefore desirable for nutrition interventions to target as young of an age group as possible [42]. By tailoring our intervention solely to third grade students, we were able to assess the cognitive abilities of this specific younger age group. Through the pre- and post-tests, we were able to quantitatively examine third graders’ ability to retain knowledge regarding nutrition and active lifestyles. As the students showed relatively few problems during test-taking and an overall improvement in the post-tests, we can conclude that children as young as 8 years old can potentially be deemed cognitively capable of receiving health education interventions in a school setting.

The second aspect of our unique contribution to child obesity intervention literature is our interdisciplinary and interprofessional approach. The wide range of the undergraduate health and pre-health students’ backgrounds provided our team with special advantages during the development of our program [43]. In our pilot study, there were four major disciplines involved (via the health and pre-health students’ backgrounds): nursing, health care management, public health, and basic sciences, and each of these fields provided unique insight into the creation of our study. Nursing knowledge was useful in designing the curriculum, particularly for the PowerPoint slides in the education presentation. Health care management and public health knowledge were essential in developing the overall structure of the intervention, including the questionnaires. Lastly, a background in pre-medical studies was helpful in interpreting the results and applying them to an educational context. By integrating all of these fields, our pilot study had an effective and comprehensive approach which incorporates inputs from all major health science fields including nursing, medicine, basic science, public health policy, and healthcare management, thereby giving undergraduate students the opportunity to engage in interprofessional collaboration in the implementation of this pilot intervention.

The third unique contribution of this study is our demonstration of the use of an intervention implemented by an interprofessional collaboration, which resulted in the extensive involvement and leadership roles undertaken by undergraduate health and pre-health students. Since involvement in research projects often begins at the undergraduate level, these years are the prime time for health science students to become actively involved in applying their learned knowledge in the community. With regards to obesity, student and registered nurses can sometimes have negative perceptions of obesity [44]. By integrating obesity education and intervention with research, health and pre-health students could further understand the etiology of obesity while cultivating more positive attitudes. In addition, by integrating the four aforementioned fields, undergraduate students were able to benefit both from utilizing their own pre-existing knowledge and from collaborating with other health science students from different academic areas, thereby achieving the goals of interprofessional education and training [2]. Such exposure serves not only to allow undergraduate students to explore other areas of research, but also to better inform them of the impact they can have on the community via a collaborative effort with professionals in other healthcare disciplines. From our experience in this study, we propose that undergraduate years are the ideal time for interdisciplinary, interprofessional, and community-based initiatives to be introduced into nursing, health, and pre-health education curricula.

## Limitations

Due to the pilot nature of our study, the data is limited by the small item size on the questionnaires. While significant improvement can be observed in the nutrition-focused questions after both interventions, no significant improvement was found in the active lifestyle and exercise-based questions after the first intervention. The latter could be the result of the fact that our intervention was a classroom-based presentation. Perhaps an intervention supplemented with physical exercise will help children retain more information regarding exercise and its role in healthy lifestyles [45]. It is also possible the small item size (15 questions) was not sufficient to yield significantly improved results. In future larger-scale implementations, questionnaires with more even distribution of nutrition and active lifestyle questions should be developed. Furthermore, because this pilot intervention is the first implementation of this questionnaire, information on external reliability is not available.

Our study also only assessed children’s knowledge of nutrition and exercise based on the food pyramid and not their lifestyle practices. Even though we have shown that the children are able to retain the knowledge on the basis of a few months, potential observational studies can also be implemented in the future to assess whether children’s knowledge about nutrition and healthy lifestyle actually translates into practice in their daily lives. Family involvement is also likely an important aspect of nutritional and lifestyle practices [18], but it was not accounted for in this pilot intervention and should be considered in future studies. In addition, since the time of the intervention, the United States Department of Agriculture has since replaced MyPyramid with MyPlate, which offers a more visual representation of dietary guidelines [38]. This new visual guide may help children in the implementation of healthy eating, but due to the pilot nature of our study, we were unable to test for this outcome.

Lastly, our two follow-ups were conducted within different timeframes after the first and second intervention. Ideally, we would have liked to conduct a follow up 6 months after the second intervention, as we did for the first intervention. This would have enabled us to compare the retention rates after the first and second round intervention. However, the school’s academic timeline prevented this possibility because at the time of our 2-monthst post-second intervention follow up, the children were ready to begin their summer vacations. Waiting any longer to conduct the follow up would have resulted in the dispersion of the children into different classes in the following school year, making tracking impossible since we did not collect any identifying information on the subjects.

## Conclusions

Despite the above limitations, the results from our interprofessional and educational health intervention still provide insight. We have demonstrated that 3^rd^ grade children are receptive to being taught material on nutrition and exercise from a short 1-hour intervention and are capable of retaining this knowledge over a course of a few months. Additionally, an intervention program as short as one hour every few months stand to make significant impact on children’s knowledge about proper nutrition and healthy lifestyles. Our results suggest the potential benefits of further expanding the short-term intervention into a longer-term community-based curriculum targeting a younger age group than commonly practiced in previous interventions.

While our interventions were not as comprehensive as those relating to the CATCH Program, which includes modifications to school food options and increased physical activity [29], through our unique incorporation of undergraduates as facilitators, we have shown that health and pre-health students can potentially be valuable resources in developing and delivering school health education programs to young elementary school students in the community. Perhaps programs such as the CATCH Program can potentially benefit from collaborations with local education institutions and involve interprofessional undergraduates. As combating childhood obesity is becoming one of the most pressing health concerns facing our society, we highly recommend the inclusion of the underutilized resource of health and pre-health students in community-based health educational interventions. Community-based partnerships with nursing institutions and inclusion of health and pre-health students in the process can potentially alleviate the financial and time constraints faced by most school-based health education programs.

Our interprofessional pilot project also benefitted the undergraduate students involved by enriching their own health and pre-health education and broadening their interdisciplinary experience in community research and intervention. Through our personal experiences and enrichment throughout the development and execution of the study, we strongly recommend health and pre-health programs to develop a curriculum in which interprofessional undergraduate students have the opportunity to collaborate with each other and develop their own research studies under a faculty mentor to facilitate health knowledge dissemination in the community.

## Figures and Tables

**Table 1:** Nutrition and Health Education Intervention Objectives.

1. Students will be able to identify all the food groups of the food pyramid
2. Students will be able to identify which foods belong to which groups of the food pyramid
3. Students will be able to identify the health benefits of foods from each of the categories of the food pyramid
4. Students will be able to recognize the importance of exercising on a regular basis
5. Students will be able to identify various ways of exercising

**Table 2:** Children’s ex-ante knowledge of nutrition and healthy lifestyle: frequency distribution of pre-test 1 responses (correct answers are in bold).

Objective	Question	Response Choices	Fre (%)
Nutrition	French fries are unhealthy because they…	**a. Are high in fats and oils**	**45 (88%)**
		b. Are made of potatoes	3 (6%)
		c. Contain a lot of sugar	2 (4%)
		d. Taste good	1 (0.02%)
	Vegetables can particularly help decrease the risk for…	a. Stomach aches	7 (14%)
		**b. Heart (cardiovascular) problems**	**29 (57%)**
		c. Headaches	4 (8%)
		d. Becoming tired quickly	11 (22%)
	Oranges are high in…	a. Vitamin A	7 (13%)
		b. Vitamin B	6 (12%)
		**c. Vitamin C**	**34 (67%)**
		d. Vitamin D	4 (8%)
	What food or drink in high in calcium?	a. Yogurt and milk	26 (51%)
		b. Cheese	3 (6%)
		c. Crackers	2 (4%)
		**d. A and B**	**20 (39%)**
	What food is high in protein?	a. Chicken and beef	16 (31%)
		b. Tofu	5 (10%)
		c. Beans	20 (39%)
		**d. All of the above**	**10 (20%)**
	Which protein below is the least healthy?	a. Green beans	2 (4%)
		b. Eggs	4 (8%)
		**c. Bacon**	**40 (78%)**
		d. All of the above are healthy	5 (9.8%)
	The brain is important for studying.	a. Candy	2 (4%)
	What food is good for the brain?	b. Crackers	12 (24%)
		**c. Fish**	**34 (67%)**
		d. Pepperoni pizza	3 (6%)
	We all know that carrots are good vegetables to eat.	a. Throat	0 (0%)
	They are particularly good for your…	b. Teeth	6 (12%)
		c. Muscles	12 (24%)
		**d. Eyes**	**32 (63%)**
	All of the following are considered junk food EXCEPT	a. Soda	7 (14%)
		**b. Milk**	**40 (78%)**
		c. Fries	2 (4%)
		d. Potato chips	1 (2%)
	Protein helps build strong________	Correct: **Muscles**	**34 (67%)**
	Calcium helps build strong________	Correct: **Bones**	**24 (47%)**
	What is one possible reason people are obese?	a. They make healthy food choices	22 (43%)
	Exercise & Healthy Lifestyles	b. They exercise on a regular basis	5 (10%)
		**c. They have obese parents**	**6 (12%)**
		d. None of the above	18 (35%)
	Exercise is good for…	a. Preventing illness	20 (39%)
		b. Helping you do better in school	5 (10%)
		c. Sleeping better	7 (14%)
		**d. All of the above**	**18 (36%)**
	All of these are reasons why exercise is good EXCEPT	a. Makes your heart healthier	4 (8%)
		b. Makes your lungs healthier	2 (4%)
		c. Helps you keep a healthy weight	7 (14%)
		**d. Makes you tired and not concentrate as well in school**	**35 (71%)**
	Exercise can make your body…	**a. Stronger and more flexible**	**47 (92%)**
		b. Weaker	0 (0%)
		c. Heavier	1 (2%)
		d. Get tired more easily	2 (4%)

**Table 3:** Percentage of students correctly answered questions on the pre- and post-tests.

Objective	Question	Pre 1	Post 1	Post 2	OR^a^ (95%) IC	OR^b^ (95%) IC
		(n=51)	(n=47)	(n=46)	Post 1 to Pre 1	Post 2 to Pre 1
Nutrition						
	French fries are unhealthy because they…	88	91	98*	1.38 (0.07,27.16)	6.00 (0.69, 51.87)
	Vegetables can particularly help decrease the risk for…	57	72*	80*	1.94 (0.56, 6.75)	3.12 (1.25, 7.79)
	Oranges are high in…	67	94*	96*	7.72 (0.64,92.35)	11.00 (2.38, 59.90)
	What food or drink in high in calcium?	41	49	54	1.38 (0.45, 4.27)	1.70 (0.76, 1.90)
	What food is high in protein?	20	30	55*	1.71 (0.38, 7.78)	4.88 (1.98, 12.04)
	Which protein below is the last healthy?	78	79	80	1.06 (0.21, 5.49)	1.13 (0.42, 3.04)
	What food is good for the brain?	67	83*	89*	2.40 (0.50, 11.55)	4.10 (1.37, 12.27)
	Vegetables are particularly good for your …	63	81*	91*	2.50 (0.58, 10.82)	6.23 (1.93, 20.13)
	All of the following are considered junk food EXCEPT…	78	87*	85	1.89(0.26, 13.80)	1.53 (0.54, 4.36)
	Protein helps build strong________	69	77	83	1.50 (0.36, 6.25)	2.17 (0.83, 5.70)
	Calcium helps build strong________	45	66*	70*	2.37 (0.73, 7.69)	2.78 (1.21, 6.42)
Exercise						
	What is one possible reason people are obese?	14	30*	65*	2.63 (0.46, 15.05)	11.79 (4.33, 32.11)
	Exercise is good for…	35	36	64*	1.04 (0.31, 3.51)	3.13 (1.36, 7.17)
	All of these are reasons why exercise is good EXCEPT	71	64	74	0.73 (0.20, 2.59)	1.18 (0.48, 2.88)
	Exercise can make your body…	92	91	93	0.88 (0.02, 31.23)	1.22 (0.26, 5.77)

a Odds ratio comparing the odds of answering correctly between post-test 1 and pre-test 1 after the first-round intervention.

b Odds ratio comparing the odds of answering correctly between post-test 2 and pre-test 1 after the second-round intervention.

* indicates p<0.05 comparing respective post-test to pre-test 1 by Student *t*-test.

**Table 4a:** Percentage of students correctly answered questions at 6 months post-1^st^ intervention: retention after 1^st^ intervention.

Objective	Question	Post 1	Pre 2	OR^a^ (95%) IC
		(n=47)	(n=47)	Post 1 to Pre 1
Nutrition				
	French fries are unhealthy because they…	91	96	2.09 (0.36, 12.02)
	Vegetables can particularly help decrease the risk for…	72	57	0.52 (0.22, 1.22)
	Oranges are high in…	94	94	0.20 (0.05, 0.76)
	What food or drink in high in calcium?	49	53	0.84 (0.38, 1.90)
	What food is high in protein?	30	53	3.80 (1.61, 8.96)
	Which protein below is the last healthy?	79	79	1.85 (.61, 5.58)
	What food is good for the brain?	83	87	1.00 (0.34, 2.93)
	Vegetables are particularly good for your …	81	89	1.99 (0.61, 5.58)
	All of the following are considered junk food EXCEPT…	87	85	0.84 (0.26, 2.71)
	Protein helps build strong________	77	57*	0.41 (0.17, 1.00)
	Calcium helps build strong________	66	60	0.76 (0.33, 1.76)
Exercise				
	What is one possible reason people are obese?	30	64*	1.90 (0.81, 4.45)
	Exercise is good for…	36	64*	3.12 (1.34, 7.22)
	All of these are reasons why exercise is good EXCEPT	64	83*	2.76 (1.05, 7.26)
	Exercise can make your body…	91	94	1.36 (0.29, 6.46)

a Odds ratio comparing the odds of answering correctly between pre-test 2 and post-test 1 at 6 months after the 1^st^-round intervention.

* indicates p<0.05 comparing pre-test 2 and post-test 1 by Student *t*-test.

**Table 4b:** Percentage of students correctly answered questions at 6 months post-1st intervention: retention after 1^st^ intervention.

Objective	Question	Post 2	Pre 3	OR^a^ (95%) IC
		(n=46)	(n=34)	Pre 3 to Post 2
Nutrition				
	French fries are unhealthy because they…	98	97	0.73 (0.04, 12.16)
	Vegetables can particularly help decrease the risk for…	80	62	0.39 (0.14, 1.07)
	Oranges are high in…	96	97	1.50 (0.13, 17.25)
	What food or drink in high in calcium?	54	68	1.76 (0.07, 4.42)
	What food is high in protein?	54	50	0.84 (0.35, 2.04)
	Which protein below is the last healthy?	80	79	0.94 (0.31, 2.83)
	What food is good for the brain?	89	88	0.92 (0.23, 3.70)
	Vegetables are particularly good for your …	89	88	0.44 (0.12, 1.72)
	All of the following are considered junk food EXCEPT…	85	85	1.04 (0.30, 3.61)
	Protein helps build strong________	83	74	0.59 (0.20, 1.72)
	Calcium helps build strong________	70	62	0.71 (0.28, 1.16)
Exercise				
	What is one possible reason people are obese?	65	50	0.53 (0.22, 1.32)
	Exercise is good for…	63	47	0.52 (0.21, 1.28)
	All of these are reasons why exercise is good EXCEPT	74	74	0.98 (0.36, 2.68)
	Exercise can make your body…	93	88	0.52 (0.11, 2.51)

a Odds ratio comparing the odds of answering correctly between pre-test 2 and post-test 1 at 6 months after the 1^st^-round intervention.

* indicates p<0.05 comparing pre-test 2 and post-test 1 by Student *t*-test.
